# Hypoparathyroidism: an uncommon adverse effect of treatment with durvalumab

**DOI:** 10.1530/EO-22-0047

**Published:** 2022-09-22

**Authors:** Alexander Kreze, Matěj Homer, Tereza Barešová, Kristina Klemperová

**Affiliations:** 1 Endocrine Clinic of Internal Medicine Department, The Bulovka University Hospital, Praha, Czech Republic; 2 Department of Internal Medicine, The Bulovka University Hospital, Praha, Czech Republic; 3 Department of Biochemistry, The Bulovka University Hospital, Praha, Czech Republic

**Keywords:** immune checkpoint inhibitors, durvolumab, immune-related adverse effects, hypoparathyroidism

## Abstract

**Summary:**

Immune checkpoint inhibitors (ICIs) are monoclonal antibodies approved for the treatment of numerous cancer types. Toxicities induced by ICIs may affect any organ system and manifest as endocrinopathy. The main side effects related to treatment are immune-related adverse events (irAEs), especially thyroid dysfunction and hypophysitis. Rare endocrine irAEs are diabetes insipidus, hypoparathyroidism, thyrotoxic crisis and hypogonadism. We report a case of hypoparathyroidism induced by ICI treatment with durvalumab, which has not previously been described.

**Learning points:**

- Treatment with immune checkpoint inhibitors (ICIs) is associated with many endocrine side effects.
- It is recommended that patients treated with ICIs are observed by an endocrinologist.
- If side effects are treated accordingly, ICI therapy can continue.

## Background

Immune checkpoints are small molecules (cytotoxic T lymphocyte antigen (CTLA-4), programmed cell death protein 1 (PD-1), programmed cell death ligand-1 (PD-L1)) expressed by immune cells involved in immune homeostasis. The targeted monoclonal antibodies directed against these regulatory checkpoint molecules (immune checkpoint inhibitors (ICIs)) are used to treat some types of cancer. PD-1 is an immune inhibitory receptor expressed on activated T cells, B cells, macrophages and natural killer cells. It has two binding ligands PDL-1 and PDL-2 expressed on normal cells. Binding of PD-1 to its ligand PD-L1 triggers an inhibitory signal, leading to reduced T-cell proliferation, inhibited T-cell activity and immune response and antitumor immunity. Physiologically, the PD-1/PD-L1 pathway emerges as a result of the need to control the degree of inflammation at locations expressing the antigen, in order to secure normal tissue from damage, prevent immune cell activation and kill normal cells. However, some malignant tumors take advantage of this mechanism. They overexpress a large number of PDL-1 on the surface to reduce T-cell activation and antigen-specific T-cell immune response and thereby bypass immune surveillance.

The mechanism of action of PD-1 and PD-L1 inhibitors is in their ability to block PD-L1 binding to PD-1, allowing T cells to then be able to kill tumor cells. ICI therapy has shown antitumor efficacy, and today it is a standard treatment for many tumor types (Deligiannis *et al.* 2021). The ICIs that are approved by the US Food and Drug Administration include anti-CTLA-4 (ipilimumab), anti-PD-1 (nivolumab, pembrolizumab and cemiplimab) and anti-PDL-1 (avelumab, atezolizumab and durvalumab) agents. While it is not fully understood why endocrine tissue is particularly vulnerable, hypotheses have been proposed. These include the expression of CTLA-4 in pituitary tissue and the role of PD-1/PD-L-1 in immune tolerance disruption during the pathogenesis of autoimmune endocrinopathy. Additionally, endocrine tissue is nonregenerative and very low volume; therefore, immune destruction has significant consequences on essential hormone secretion (Hattersley *et al.* 2021).

## Case presentation

The report describes the case of a 70-year-old man with history of primary non-small cell lung cancer in the upper lobe of the left lung (T4N2M0) for which he received previous treatment involving a combination of radiotherapy and chemotherapy (cumulative dose of 54 Gy and three cycles of cisplatin and pemetrexed). The patient received maintenance immunotherapy with 1500 mg of durvalumab administered twice, 4 weeks apart. Within a month of the last dose of durvalumab, the patient developed nausea, generalized weakness, muscle pain with fasciculations and carpopedal spams.

Upon presentation at our hospital, the patient reported no history of autoimmune endocrinopathy, surgery or radiotherapy on the neck. His physical examination was positive for Chvostek and Trousseau signs, but muscle fasciculations at rest were unremarkable. Laboratory analyses upon admission indicated severe hypocalcemia, hyperphosphatemia, a low level of serum 25-hydroxyvitamin D and an undetectable parathyroid hormone (PTH). The laboratory tests are summarized in Table 1.

**Table 1:** Laboratory analysis upon admission.

Test	Patient level	Normal range
Calcium	1.41 mmol/L	2.1–2.55 mmol/L
Ionized calcium	0.65 mmol/L	1.13–1.32 mmol/L
Phosphate	2.11 mmol/L	0.74–1.52 mmol/L
Albumin	43 g/L	32.0–46.0 g/L
Magnesium	0.69 mmol/L	0.66–1.07 mmol/L
25-hydroxyvitamin D	38.6 nmol/L	50.0–125.0 nmol/L
PTH	<0.58 pmol/L	1.58–6.03 pmol/L

PTH, parathyroid hormone.

A 1,25-dihydroxyvitamin D level was not measured.

The patient´s treatment commenced with i.v. calcium chlorate (273 mg of elemental calcium) three times daily, 0.25 μg of calcitriol twice daily because of unmeasurable PTH and presumed resultant impairment of 1 alpha hydroxylation of 25 hydroxyvitamin D and 0.25 mg of hydrochlorothiazide once daily. After 6 days of this regimen, the patient´s serum calcium level was 1.86 mmol/L, ionized calcium was 0.93 mmol/L and PTH remained undetectable (<0.58 pmol/L). On the seventh day, the patient was discharged on daily regimen of calcium carbonate (equivalent to 1000 mg of elemental calcium), 0.5 µg calcitriol and 0.25 mg hydrochlorothiazide. After 1 month on this therapy, the patient´s serum calcium was 2.07 mmol/L; the patient was also free of other symptoms and continued treatment with durvalumab.

## Discussion

ICIs are approved for the treatment of some types of advanced cancer. Toxicities induced by ICIs are autoimmune and referred as immune-related adverse events (irAEs). The incidence of endocrinopathies during ICI therapy reaches 10% in meta-analysis of 38 studies, which involved a total of 7551 patients under ICIs (Deligiannis *et al.* 2021). Among the patients on anti-CTLA-4 agent monotherapy, the most frequent endocrine irAE is hypophysitis (5.6%), whereas among patients on anti-PD-1/PD-L1 agent monotherapy, the most frequent irAE is hypothyroidism (8.5%). Rare endocrinopathies during ICI therapy are diabetes mellitus, hypogonadotropic hypogonadism in the absence of hypophysitis, diabetes insipidus, syndrome of inappropriate antidiuretic hormone secretion (SIADH) and transient adrenocorticotrophic hormone (ACTH)-dependent hypercortisolaemia (George *et al.* 2021).

Hypoparathyroidism is an extremely rare irAE. Hypoparathyroidism should be considered when hypocalcemia is associated with low-normal or low PTH levels. Antiparathyroid and alcium-sensing receptor-activating autoantibodies may be detectable in patients with ICI-related hypoparathyroidism mediated by these autoantibodies (George *et al.* 2021). Acute symptomatic hypoparathyroidism develops between 3 weeks and 7 months of treatment with ICIs (Umeguchi *et al.* 2018, El Kawkgi *et al.* 2020). Hypoparathyroidism as a complication in checkpoint inhibitor therapy is largely irreversible, and long-term treatment with an activated form of vitamin D analog and calcium supplements is required.

Hypoparathyroidism is a rare complication induced by ICIs and encountered in treatment with nivolumab (Edd *et al.* 2018, Piranavan *et al.* 2019), ipilimumab with nivolumab (Cubb *et al.* 2017, Win *et al.* 2017, Trinh *et al.* 2019, Dadu *et al.* 2020, El Kawgki *et al.* 2020) and pembrolizumab (Umeguchi *et al.* 2018, Lupi *et al.* 2020). In the three reported cases, other concomitant endocrinopathies were confirmed: autoimmune thyroiditis with a thyrotoxic phase (Win *et al.* 2017, Dadu *et al.* 2020) and hypophysitis with adrenal insufficiency (El Kawgki *et al.* 2020). Despite similarities in clinical manifestation, emerging data suggest immunological differences between irAEs and ‘traditional’ autoimmune diseases, as conventional autoantibodies are often not detectable when irAEs are diagnosed (Khan & Monzon *et al.* 2020). Mechanisms which generate irAEs have been suggested: preexisting susceptibility to autoimmunity, aberrant presentation of ‘self’ by the tumor, loss of tolerance driven by the tissue or tumor microenvironment (Burke *et al.* 2020).

All reported cases of ICI-induced hypoparathyroidism in the Embase and Medline databases are listed in Table 2.

**Table 2:** All reported cases of ICI-induced hypoparathyroidism.

Authors	Drug diagnosis	Onset hypoparathyroidism after treatment ICI	Duration hypoparathyroidism	Antibodies
Cubb *et al*.	I + N Melanoma	4 weeks	Parathyroid function has not recovered since diagnosis	Negative anti-PTH antibodies
Dadu *et al*.	I + N Melanoma	4 weeks	3 years and 3 months	Negative CaSR antibodies
Edd *et al*.	N NSCLC	5 months	Passing hospice shortly after moving	Not measured
El Kawkgi *et al*.	I + N Melanoma	7 months	77 days	Negative anti-PTH antibodies
Lupi *et al*.	P Lung adenocarcinoma	15 weeks	9 months	Positive CaSR antibodies
Piranavan *et al*.	N SCLC	5 months	1 year	Positive CaSR antibodies
Trinh *et al*.	I + N Melanoma	4 weeks	Improvement after 8 months	Detectable CaSR antibodies
Umeguchi *et al*.	P NSCLC	3 weeks	5 months	Not measured
Win *et al*.	I + N Melanoma	1,5 months	4 months	Not measured

CaSR, calcium-sensing receptor; I, ipilimumab; ICI, immune checkpoint inhibitor; N, nivolumab; NSCLC, non-small cell lung cancer; P, pembrolizumab; PTH, parathyroid; SCLC, small cell lung cancer.

Because of the lack of associated causes of hypoparathyroidism (anterior neck surgery, radiotherapy of the neck, absence of other autoimmune syndromes, negative antiparathyroid antibodies, hypomagnesemia, severe vitamin D deficiency and hypoalbuminemia), we believe that the patient developed hypoparathyroidism as a very rare complication associated with durvalumab therapy.

## Conclusion

ICIs are effective therapeutic modalities for many cancers, but their use also produces many adverse side effects in the endocrine system. Supervision by an endocrinologist is therefore recommended for optimal care. Patients on ICIs should be routinely monitored for the development of endocrinopathies before immunotherapy: fasting venous glycemia (if anti-PD1/PD-L1), natremia, thyroid-stimulating hormone (TSH), fT4, 08:00  h cortisol (without corticosteroid intake), +/− ACTH (depending on 08:00 h cortisol level), luteinizing hormone (LH), follicle-stimulating hormone (FSH), testosterone in males, LH, FSH, estradiol in females and FSH in menopausal females. During immunotherapy, patients should be monitored in each course of treatment for 6 months and every two courses for the following 6 months. Patients should also be monitored in cases of clinical alert signs: fasting venous glycemia (if anti-PD1/PD-L1), natremia, TSH, fT4, 08:00  h cortisol and testosterone in males (Castinetti *et al.* 2019). In contrast to other irAEs, patients even with high grades of endocrine irAEs may continue their ICI therapy, provided the hormone replacement therapy is adequate and the symptoms are controlled (George *et al.* 2021).

## Declaration of interest

The authors declare that there is no conflict of interest that could be perceived as prejudicing the impartiality of this case report.

## Funding

This work did not receive any specific grant from any funding agency in the public, commercial or not-for-profit sector.

## Patient consent

Written informed consent for publication of their clinical details was obtained from the patient.

## Author contribution statement

A K conceived the study, A K and M H did the literature search, analyzed data and wrote the manuscript, T B was in-patient treating doctor, K K contributed to biochemistry, immunology and hormonal analysis and interpretations. All contributors approved the final accepted version of the manuscript.

## References

[bib1] Burke KP Grebinoski S Sharpe AH Vignali DAA 2020 Understanding adverse events of immunotherapy: a mechanistic perspective. Journal of Experimental Medicine 1 e20192179. (10.1084/jem.20192179) PMC7754677 33601411

[bib2] Castinetti F Albarel F Archambeaud F Bertherat J Bouillet B Buffier P Briet C Cariou B Caron P Chabre O 2019 French Endocrine Society Guidance on endocrine side effects of immunotherapy. Endocrine-Related Cancer 26 G1–G18. (10.1530/ERC-18-0320) 30400055 PMC6347286

[bib3] Cubb T Patel S 2017 Primary hypoparathyroidism: a new endocrine immune related adverse event (irAEs) secondary to combination treatment with PD-1 and CLA-4 checkpoint inhibitors. Endocrine Reviews 38 (Supplement 1). (10.1093/edrv/38.supp.1)

[bib4] Dadu R Rodgers TE Trinh VA Kemp EH Cubb TD Patel S Simon JM Burton EM Tawbi H 2020 Calcium-sensing receptor autoantibody-mediated hypoparathyroidism associated with immune checkpoint inhibitor therapy: diagnosis and long-term follow-up. Journal for ImmunoTherapy of Cancer 8 e000687. (10.1136/jitc-2020-000687) PMC7319718 32581059

[bib5] Deligiannis NG Sosa S Danilowicz K Rizzo LFL 2021 Endocrinne dysfunction induced by immune checkpoint inhibitors. Medicina 81 269–278. 33906146

[bib6] Edd T Taufeeq M Buschur E 2018 Nivolumab induced hypoparathyroidism. Endocrine Reviews 39 (Supplement 1). (10.1093/edrv/39.supp.1)

[bib7] El Kawkgi OM Li D Kotwal A Wermers RA 2020 Hypoparathyroidism: an uncommon complication associated with immune checkpoint inhibitor therapy. Mayo Clinic Proceedings 4 821–825. (10.1016/j.mayocpiqo.2020.07.006) 33367219 PMC7749243

[bib8] George AS Fernandez CJ Eapen D Pappachan JM 2021 Organ-specific adverse events of immune checkpoint inhibitor therapy, with special reference to endocrinopathies. TouchREVIEWS in Endocrinology 17 21–32. (10.17925/EE.2021.17.1.21) 35118443 PMC8320015

[bib9] Hattersley R Nana M Lansdown AJ 2021 Endocrine complications of immunotherapies: a review. Clinical Medicine 21 e212–e222. (10.7861/clinmed.2020-0827) 33762389 PMC8002767

[bib10] Khan OF Monzon J 2020 Diagnosis, monitoring, and management of adverse events from immune checkpoint inhibitor therapy. Current Oncology 27 S43–S50. (10.3747/co.27.5111) 32368173 PMC7194002

[bib11] Lupi I Brancatella A Cetani F Latrofa F Kemp EH Marcocci C 2020 Activating antibodies to the calcium-sensing receptor in immunotherapy-induced hypoparathyroidism. Journal of Clinical Endocrinology and Metabolism 105 1581–1588. (10.1210/clinem/dgaa092) 32112105

[bib12] Piranavan P Li Y Brown E Kemp EH Trivedi N 2019 Immune checkpoint inhibitor-induced hypoparathyroidism associated with calcium-sensing receptor-activating autoantibodies. Journal of Clinical Endocrinology and Metabolism 104 550–556. (10.1210/jc.2018-01151) 30252069

[bib13] Trinh B Sanchez GO Herzig P Läubli H 2019 Inflammation-induced hypoparathyroidism triggered by combination immune checkpoint blockade for melanoma. Journal for ImmunoTherapy of Cancer 7 52. (10.1186/s40425-019-0528-x) PMC6385398 30791949

[bib14] Umeguchi H Takenoshita H Inoue H Kurihara Y Sakaguchi C Yano S Hasuzawa N Sakamoto S Sakamoto R Ashida K 2018 Autoimmune-related primary hypoparathyroidism possibly induced by the administration of pembrolizumab: a case report. Journal of Oncology Practice 14 449–451. (10.1200/JOP.18.00076) 29906213

[bib15] Win MA Thien KZ Qdaisat A Yeung SJ 2017 Acute symptomatic hypocalcemia from immune checkpoint therapy-induced hypoparathyroidism. American Journal of Emergency Medicine 35 1039.e5–1039.e7. (10.1016/j.ajem.2017.02.048) 28363614

